# Activation of the Wnt Pathway by *Mycobacterium tuberculosis*: A Wnt–Wnt Situation

**DOI:** 10.3389/fimmu.2017.00050

**Published:** 2017-02-01

**Authors:** Tomás Villaseñor, Edgardo Madrid-Paulino, Rafael Maldonado-Bravo, Antonio Urbán-Aragón, Leonor Pérez-Martínez, Gustavo Pedraza-Alva

**Affiliations:** ^1^Departamento de Medicina Molecular y Bioprocesos, Instituto de Biotecnología, Universidad Nacional Autónoma de México, Cuernavaca, Morelos, Mexico

**Keywords:** tuberculosis, Wnt signaling, macrophage infection, inflammation, immune response, microRNAs

## Abstract

*Mycobacterium tuberculosis* (*M. tuberculosis*), an intracellular pathogenic Gram-positive bacterium, is the cause of tuberculosis (TB), a major worldwide human infectious disease. The innate immune system is the first host defense against *M. tuberculosis*. The recognition of this pathogen is mediated by several classes of pattern recognition receptors expressed on the host innate immune cells, including Toll-like receptors, Nod-like receptors, and C-type lectin receptors like Dectin-1, the Mannose receptor, and DC-SIGN. *M. tuberculosis* interaction with any of these receptors activates multiple signaling pathways among which the protein kinase C, the MAPK, and the NFκB pathways have been widely studied. These pathways have been implicated in macrophage invasion, *M. tuberculosis* survival, and impaired immune response, thus promoting a successful infection and disease. Interestingly, the Wnt signaling pathway, classically regarded as a pathway involved in the control of cell proliferation, migration, and differentiation in embryonic development, has recently been involved in immunoregulatory mechanisms in infectious and inflammatory diseases, such as TB, sepsis, psoriasis, rheumatoid arthritis, and atherosclerosis. In this review, we present the current knowledge supporting a role for the Wnt signaling pathway during macrophage infection by *M. tuberculosis* and the regulation of the immune response against *M. tuberculosis*. Understanding the cross talk between different signaling pathways activated by *M. tuberculosis* will impact on the search for new therapeutic targets to fuel the rational design of drugs aimed to restore the immunological response against *M. tuberculosis*.

## Introduction

**Figure 1:**
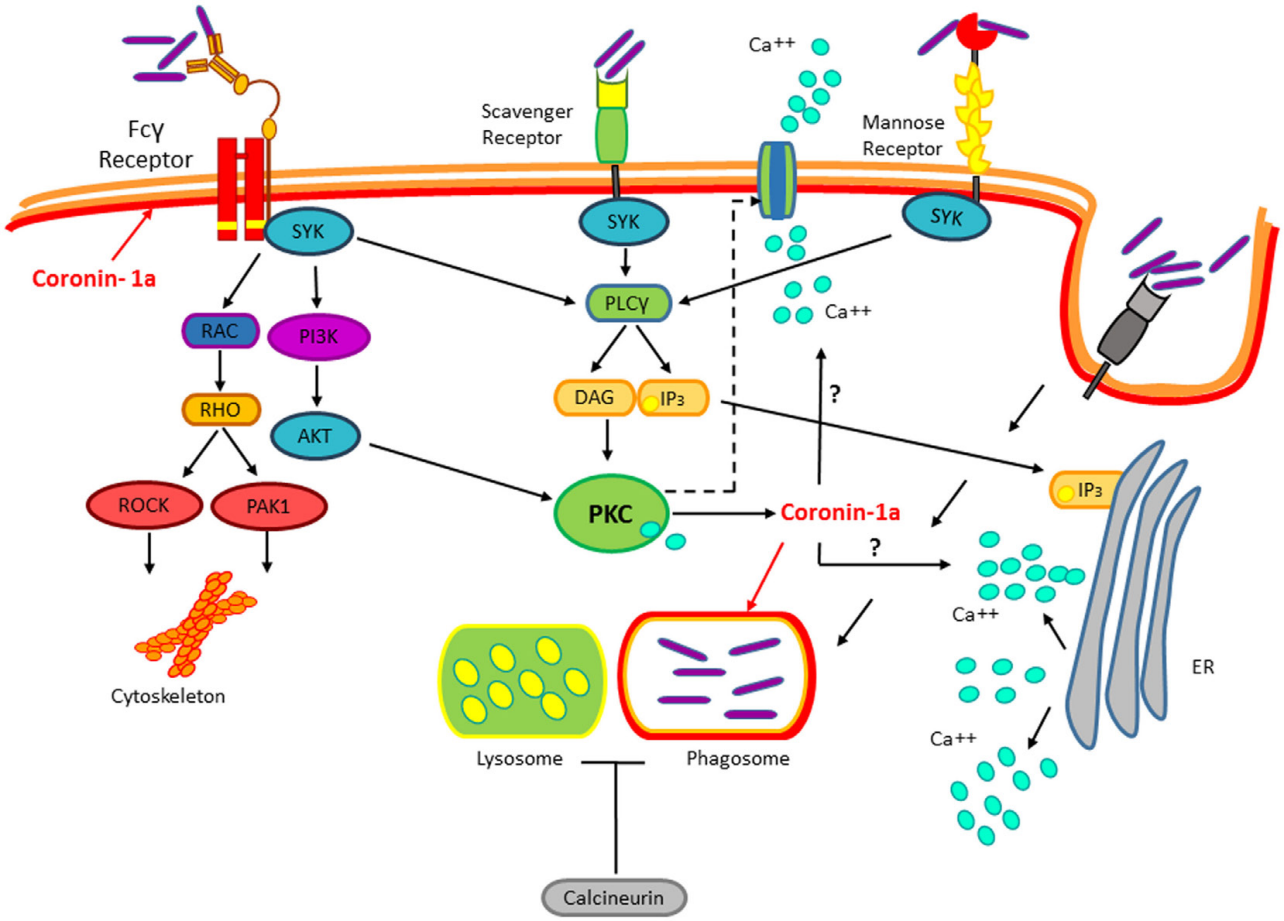
***Mycobacterium tuberculosis* (*M. tuberculosis*) recognition by immune receptors**. During the infection, several classes of PPRs such as the Fcγ receptor, the Scavengers, and Mannose receptors recognize *M. tuberculosis*. Mycobacterium interaction with these receptors activates the protein kinase C pathway. This pathway is implicated in the cytoskeletal arrangements, macrophage invasion, and *M. tuberculosis* survival. Upon invasion of the macrophage by *M. tuberculosis*, coronin-1a is recruited to the mycobacterial phagosome leading to the induction of calcium fluxes, thus resulting in calcineurin activation, which blocks the phagosome–lysosome fusion by an unknown mechanism.

*Mycobacterium tuberculosis* (*M. tuberculosis*) is the cause of human tuberculosis (TB), which is regarded as one of the most harmful pathogen, just behind HIV, that is responsible for more deaths than any other microorganism. The WHO in 2015 estimated that 1/3 of the planet’s population harbor this bacterium, yet only 2–23% will develop disease during their lifespan. *M. tuberculosis* is a Gram-positive bacterium that spreads by aerosols. Upon entry into the lungs, *M. tuberculosis* infects alveolar macrophages, a process mediated by a variety of receptors expressed on the surface of these phagocytic cells (1). Receptors expressed in macrophages involved in *M. tuberculosis* internalization include complement receptors (2), C-type lectin receptors, dectin-1, mannose receptors, scavenger receptors (3–5), CD14 (6), CD43 (7), and lung surfactant protein A (8). However, results obtained from studies using null mice for the expression of any of these receptors indicate that none of them are essential for *M. tuberculosis* macrophage invasion (9). Likewise, none of these receptors confer a survival advantage for *M. tuberculosis* (10). The engagement of these receptors on the macrophage cell surface normally would result in the activation of different signaling pathways that lead to the destruction of the invading pathogen. However, the *M. tuberculosis* success as an intracellular pathogen resides in the manipulation of these signaling pathways by several mechanisms to avoid bactericidal activities of the host macrophages and survive inside the cell host (11–14). Accordingly with the fact that, most of the receptors engaged by *M. tuberculosis* upon the first contact with the macrophage activate proteins of the protein kinase C (PKC) family as well as calcium mobilization (Figure 1), among other known mechanisms involved in blocking phagosome-lysosome fusion, recently it was shown that *Mycobacterium bovis* (*M. bovis*) promotes the coating of the phagosome with coronin-1a, which leads to the induction of calcium fluxes activating the calcium-dependent phosphatase calcineurin. Upon *M. bovis* infection, coronin-1a-deficient macrophages show impaired calcium mobilization and calcineurin activation, resulting in mycobacterial destruction. Consistent with this, preventing calcium mobilization, with calcium chelators, or inhibiting calcineurin activation in wild-type macrophages reduces *M. bovis* proliferation and survival (1). Therefore, these data suggest that *M. tuberculosis*-dependent inhibition of phagosome–lysosome fusion involves the engagement of multiple receptors that lead to dephosphorylation of proteins regulating phagosome maturation (15). Nonetheless, the molecular mechanism by which coronin-1a leads to calcium mobilization or whether coronin-1a is subjected to regulation by any of the kinases activated by *M. tuberculosis* upon interaction with its plethora of receptors remain to be defined.

**Figure 2:**
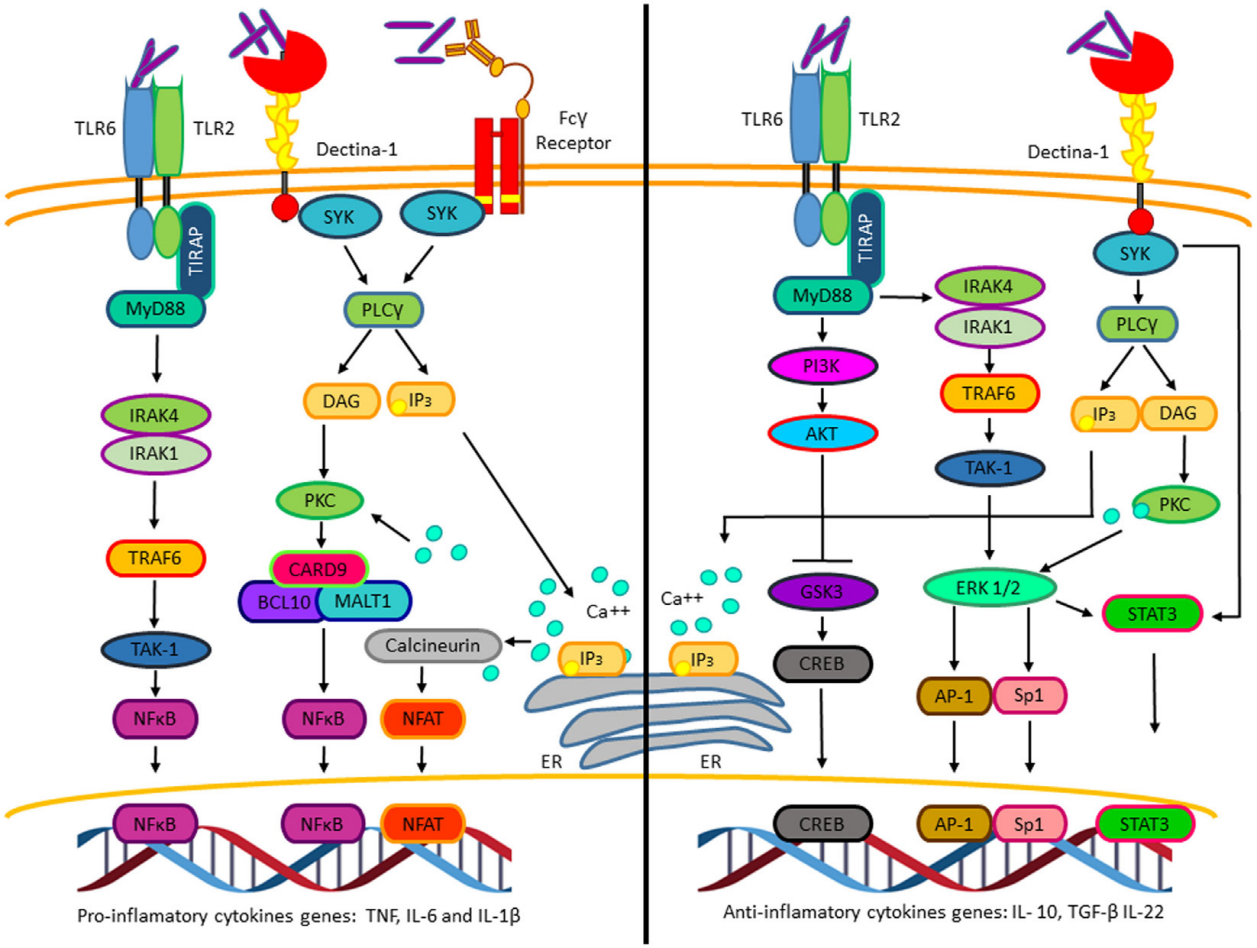
**The pro-inflammatory and anti-inflammatory cytokines’ genes activated by *Mycobacterium tuberculosis* (*M. tuberculosis*)**. Pro-inflammatory (left). Interaction of *M. tuberculosis* with TLR2, Dectin-1, and Fcγ receptors activates NFκB and nuclear factor of activated T cells, which promotes transcription of genes such as tumor necrosis factor, IL-6, and IL-1β. Anti-inflammatory (right). Interaction of *M. tuberculosis* to TLR-2 and Dectin-1 through different mechanisms induces the activation of the transcription factors CREB, AP-1, Sp1, and STAT3, which initiates the transcription of genes such as IL-10, TGF-β, and IL-22.

In addition to the signaling pathways activated by *M. tuberculosis* that allow macrophage invasion and survival, *M. tuberculosis* also activates numerous signaling pathways promoting cytokines and chemokines expression. Among those, pro-inflammatory cytokines like tumor necrosis factor (TNF), interleukin-1β (IL-1β), and interleukin-6 (IL-6) are secreted few hours after macrophage infection and precedes anti-inflammatory cytokine production, including transforming growth factor β (TGF-β) and interleukin-10 (IL-10) (Figure 2) (16). The production of the inflammatory cytokines and chemokines at early stages of the infectious process is crucial for the recruitment of neutrophils, macrophages to the lung, and later on for the recruitment of activated T cells. Depending on the final balance between pro- and anti-inflammatory cytokines, the host can either assemble a response that eliminates the infection or result in granuloma formation, thus controlling the infection without bacterial elimination (17). Different experimental evidence suggests that IL-10 expression is critical for mycobacterium survival, granuloma formation, and attenuation of the inflammatory response. Transgenic mice expressing human IL-10 specifically in the lungs showed increased bacterial burden in *Mycobacterium avium* (*M. avium*)-containing granulomas. This correlated with decreased pro-inflammatory cytokines’ levels, such as TNF and IL-12 [reviewed in Ref. (18)].

Thus, *M. tuberculosis* infection depends on the mycobacterial ability to hijack different macrophage signaling pathways, at the early stages of infection to avoid its destruction, and at the later stages of infection to alter the cytokine profile to attenuate the adaptive immune response. In addition to the well-characterized pathways involved in these processes, like the PKC, MAPK, NFκB and JAK/STATs pathways (Figures 1 and 2), cumulative experimental evidence point out a role for the Wnt/β-catenin pathway during *M. tuberculosis* infection. Here, we will discus the molecular mechanism by which *M. tuberculosis* activates the Wnt/β-catenin pathway and its role during infection.

## The Wnt Signaling Pathways

The Wnt signaling pathway has been extensively studied and reviewed (19–23). This signaling pathway is an ancient and highly conserved signaling mechanism and involves important molecular cascades that regulate cell fate throughout lifespan (19, 24). The Wnt signaling pathway has been generally associated with cellular proliferation, differentiation, apoptosis, motility, and polarization of cells, in invertebrates and mammals (25). Mutations in molecules controlling this pathway that result in constitutive activation of the pathway may lead to colon cancer, hair follicle tumors, and leukemia (26). However, the role of Wnt signaling in the immune response against pathological bacteria is poorly understood.

The Wnt/β-catenin network was first identified in 1982 with the discovery of the proto-oncogene *Int1* in mice (27). Later in 1987, the segment polarity gene wingless, the *Drosophila melanogaster* homolog of *Int1* was cloned and shown to be required for proper wing formation (28). Until now, there are three different characterized Wnt pathways: the canonical pathway or Wnt/β-catenin (cadherin-associated protein β), which involves β-catenin and members of T-cell factor (TCF)/lymphoid enhancer-binding factor (LEF) family of transcription factors (TCF/LEF); the planar cell polarity (PCP) pathway; and the Wnt/Ca^++^ pathway (29). The ligands involved in the Wnt pathway are soluble factors that can be divided into two subclasses: the Wnt1 and Wnt5a. The Wnt1 proteins (Wnt1, Wnt2, Wnt3, Wnt3a, Wnt7a, Wnt8a, and Wnt10b) (30) participate in the canonical Wnt pathway. On the other hand, the Wnt5a proteins (Wnt4, Wnt5a, and Wnt11) participate in non-canonical signaling pathway (31).

## The Canonical Pathway

**Figure 3:**
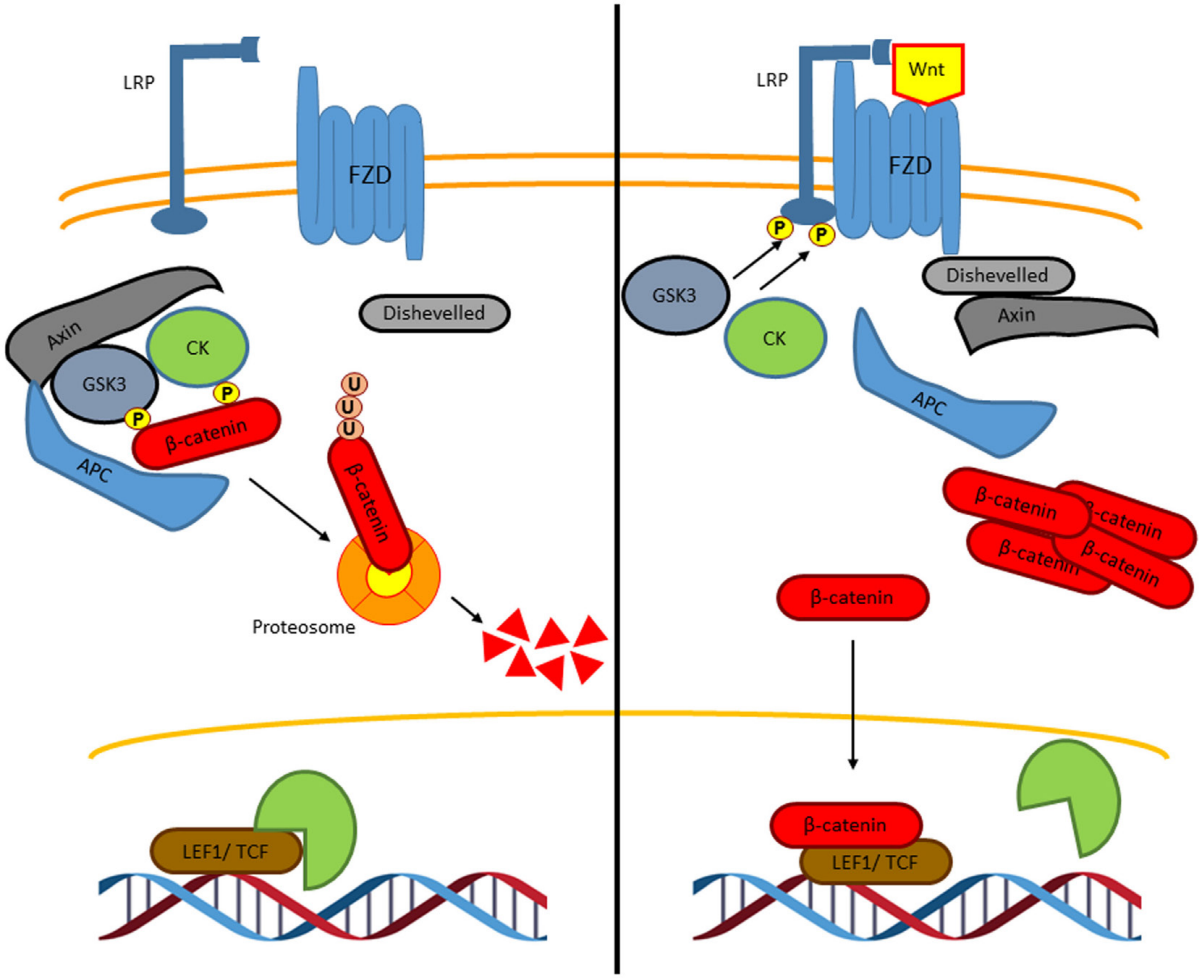
**The canonical Wnt signaling pathway**. In the absence of Wnt ligands, β-catenin interacts with a degradation complex, which is formed by Axin, APC, GSK3, and CK1. In this complex, β-catenin is phosphorylated by GSK3 and by CK1 leading to ubiquitylation and subsequently degradation of β-catenin in the proteasome. When β-catenin is degraded, Groucho, which is a co-repressor protein, interacts with LEF1 and TCF. The Groucho/LEF1/TCF complex represses the transcription of target genes (left). Wnt ligands interact with the Frizzled receptor, a G protein-coupled receptor, and with the co-receptor LRP. GSK3 and CK1 induce the phosphorylation of LRP leading to the recruitment of axin and disheveled to the LRP/G protein-coupled receptor complex releasing β-catenin. Then, β-catenin is accumulated in the nucleus where it binds to LEF1 and TCF to induce the transcription of specific genes (right).

The canonical Wnt signaling or Wnt/β-catenin signaling is the most and best understood and characterized pathway (Figure 3) (29). In the absence of Wnt ligands, cytoplasmic β-catenin is phosphorylated on its N-terminal region, first on Ser 45 by casein kinase 1 (CK1), and then on Ser33, Ser37, and Thr41 by the glycogen synthase kinase 3 β (GSK3β) to create recognition sites for β-transducin-repeat-containing protein (βTRCP), leading to the β-catenin ubiquitylation and preteasomal breakdown, preventing in this way its nuclear translocation and Wnt-dependent gene expression (32). To activate the Wnt/β-catenin pathway, the Wnt ligands bind the receptor complex formed by the Frizzled (FZD) receptor and the low-density lipoprotein receptor-related protein (LRP), leading to the recruitment of the protein disheveled (DVL). This recruitment inactivates the β-catenin destruction complex, which is conformed by protein Axin, tumor-suppressor adenomatous polyposis coli gene product (APC), CK1, and GSK3β, thus preventing β-catenin phosphorylation by GSK3 and consequently, its proteasomal degradation. This, results in β-catenin accumulation and translocation to the nucleus where it drives the expression of TCF/LEF-dependent genes [c-Myc, CyclinD-1, Axin2, metalloproteinases, CD44, peroxisome proliferator-activated receptor gamma (PPARγ, etc.)], which are involved in cell proliferation, survival, and cell differentiation (31).

## Non-Canonical Pathways

**Figure 4:**
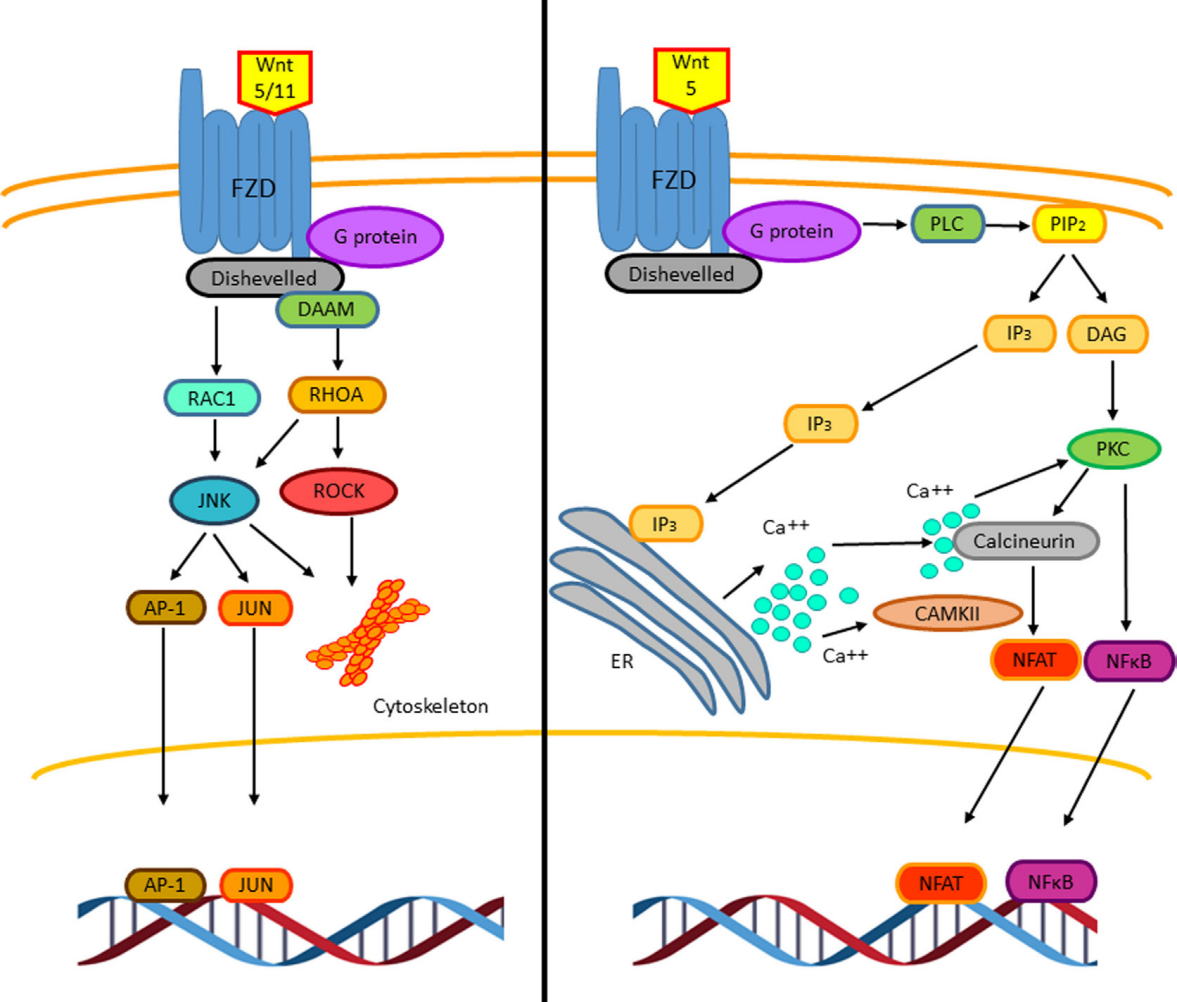
**The non-canonical Wnt signaling pathway**. Two β-catenin independent Wnt signaling pathways have been reported. The planar cell polarity (PCP) pathway is triggered by Wnt5 and Wnt11. Wnt5/11 binds Frizzled leading to the activation of a trimeric G protein that induces the activation of disheveled and DAAM. Together these proteins trigger the activation of the small GTPases RHOA and RAC-1 that leads to the activation of the kinases JUN kinase (JNK) and ROCK. These signaling pathways are involved in cell adhesion, migration, and cell cytoskeleton organization. The stress response pathway involves the activation of JNK, which phosphorylates the transcriptional factors AP-1 and JUN leading to their translocation to the nucleus to regulate gene expression (left). The Wnt/Ca^2+^ signaling pathway is triggered by Wnt5–FZD-2. The activation of this pathway is mediated through G proteins, which induces the phospholipase C activation, leading to the hydrolysis of PIP2 into DAG and IP3. The IP3 induces the release of intracellular Ca^++^, activation of calcineurin, and CAMKII. The active calcineurin induces the nuclear factor of activated T cells (NFAT) activation. Intracellular calcium as well as DAG activates protein kinase C (PKC) increasing the activity of calcineurin triggering the translocation of the transcriptional factor NFAT to the nucleus. Similarly, PKC induces the activation and translocation of NFκB to the nucleus (right).

The non-canonical Wnt pathway is defined as Wnt or FZD receptor-initiated signaling that is independent of β-catenin transcriptional function (33). So far, two different non-canonical Wnt pathways have been described, the Wnt–Ca^++^ pathway and the PCP pathway (Figure 4) (29). Such classifications are not strictly rigid and exclusive as these pathways overlap with or intersect one another (33). Wnt5 and Wnt11 participate in initializing PCP pathway through FZD and DVL (mammalian homolog of Drosophila disheveled) activating trimeric G proteins and DVL and DAAM (disheveled-associated activator of morphogenesis), which together activate the RHOA (RAS homolog gene-family member A)–ROCK (RHO-associated coiled-coil containing protein kinase) pathway, that mediates cytoskeletal re-organization (34). The DVL–DAAM–RHOA complex activates RHOA, resulting in ROCK kinase activation (33). In addition to RHOA activation, DVL also activates RAC1 (Ras-related C3 botulinum toxin substrate 1) leading to the activation of the JUN kinase stress response pathway, thus influencing cell shape (29). Hence, the PCP pathway, through regulating actin-dependent changes in cytoskeleton, controls cell adhesion and migration. Interestingly, in both T and B lymphocytes, it has been observed that this pathway can inhibit the canonical Wnt pathway by downregulating β-catenin levels (35, 36). Downstream pathways of DVL regulate the cytoskeleton and cell polarity; however, in immune cells, the molecular mechanism involved is not clear yet (29).

The Wnt–Ca^++^ signaling pathway can influence the activation of both, canonical and non-canonical Wnt pathway. It has been proposed that Wnt5 and Frizzled-2 can initiate this pathway (37, 38). The Wnt–FZD complex activates phospholipase C through a small G protein. This activation leads to cleavage of phosphatidylinositol-4-5-biphosphate (PIP2) to diacylglycerol (DAG) and inositol triphosphate (IP3). IP3 binding to its receptor leads to an increase in intracellular calcium; DAG and calcium activates different members of the PKC family. Increased calcium levels also lead to activation of the phosphatase calcineurin, promoting the activation of the nuclear factor of activated T cells (NFAT) (39, 40), that is involved in T-cell receptor-mediated activation of interleukin-2 production in T-lymphocytes (29). Another connection between the non-canonical Wnt signaling pathway and NFAT function involves GSK3β, which through NFAT phosphorylation promotes its exit from the nucleus (41) and reduces NFAT-dependent gene expression. All together, these observations suggest that the non-canonical Wnt signaling pathways regulate NFAT-dependent gene expression by different mechanisms (29, 41).

## Wnt Signaling and Inflammation

**Figure 5:**
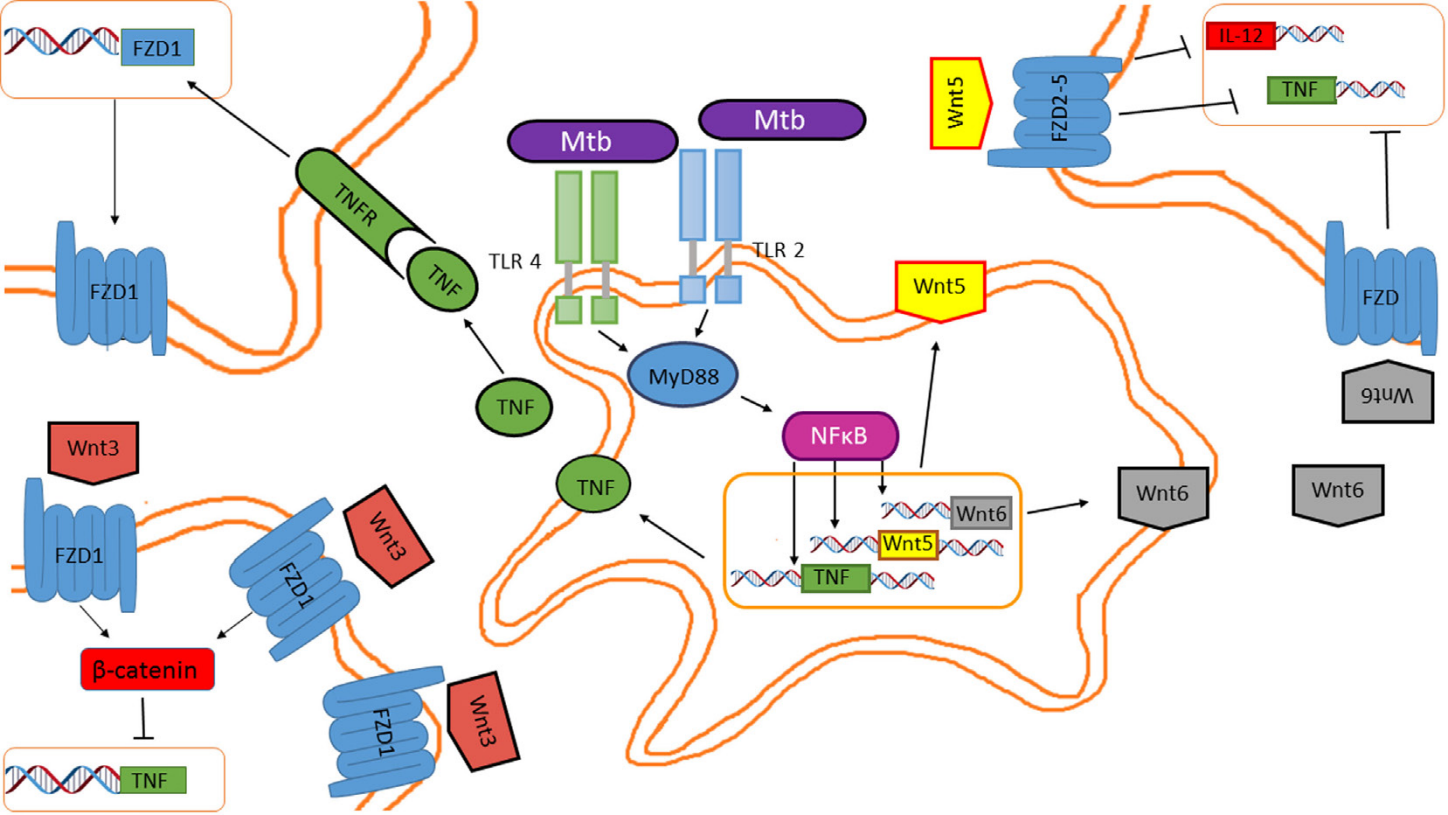
***Mycobacterium tuberculosis* (*M. tuberculosis*) inhibits tumor necrosis factor (TNF) through the Wnt pathway in a paracrine and autocrine manner**. Wnt5, Wnt6, and pro-inflammatory cytokines are induced in macrophages in response to mycobacterial infection. Secreted Wnt5 and Wnt6 are recognized by FZD receptors expressed on the cell surface of neighboring macrophages, impairing TNF and IL-12 expression. Additionally, TNF released by the *M. tuberculosis*-infected macrophages promotes *FZD1* expression in neighboring macrophages, rendering them susceptible to Wnt3 actions, which inhibits TNF expression.

Recently, the role of Wnt signaling pathway in inflammatory processes started to be understood. Wnt signaling can prevent or promote inflammation. For example, exposing 3T3-L1 preadipocytes to TNF promotes Wnt10b expression and enhances activation of the canonical Wnt pathway, resulting in altered adipocyte differentiation toward an inflammatory phenotype (42). However, whether Wnt10b mediates TNF-induced preadipocyte differentiation toward the pro-inflammatory phenotype remains to be elucidated. Mouse microglial cells upregulate the expression and release of IL-6, IL-12, and IFNγ in response to Wnt3a (43). In contrast, Wnt3a has an anti-inflammatory effect on macrophages, while Wnt11 has the same effect on mouse colonic cells (44). These data indicate that the Wnt proteins promote pro- or anti-inflammatory responses and that this depends on the cellular context, the type of insult, and the cytokine environment (Figure 5). The fact that cytokines can upregulate Wnt expression raises the question of how many of the cellular responses triggered by cytokines are mediated by a paracrine effect mediated by Wnt factors and the activation of both the canonical and non-canonical Wnt pathways?

There is very scant information regarding the role of different Wnt members in response to pathogenic bacterial infections and in particular on their role in *M. tuberculosis* infection. Table 1 summarizes our current knowledge on this matter.

**Table 1:** **The role of the Wnt family members in TB**.

Wnt homolog	Found in TB disease	Roll in TB infection	Pathway	Cellular function	Model	Receptors involved
Wnt3a	Downregulated	Anti-inflammatory prevents TB infection	Canonical	Necrosis inhibition, promotes apoptosis, inhibits migration, promotes cellular adhesion, inhibits TNF secretion in a paracrine manner	Mouse (Raw cells)	Human (THP-1 cells) (44–47)	FZD 1
Wnt5a	Downregulated/upregulated	Anti-inflammatory promotes TB infection	Non-Canonical	Promotes phagocytosis, inhibits phagolysosome formation, inhibits IL-12 and INFγ production	Mouse (Raw cells), human (48, 49)	FZD 2,4,7
Wnt6	Upregulated	Anti-inflammatory promotes TB infection	Non-canonical	Inhibits TNF production, promotes the formation of foamy cells, cellular proliferation, and M2 phenotype	Mouse (50)	Unknown
Wnt1, Wnt2, Wnt4, Wnt7, Wnt8, Wnt10b	Downregulated	–	–	–	Mouse (50)	FZD 1,3,4,5,7,8
Wnt10a	Upregulated	–	–	–	Mouse (50)	Unknown

*Wnt, wingless/Int factor; TB, tuberculosis; TNF, tumor necrosis factor; IL-12, interleukin-12; INFγ, interferon γ; M2, macrophage alternative activation; FZD, frizzled*.

Additionally, the transcription factors that regulate the gene expression of the distinct Wnt and FZD family members in response to cytokines are largely unknown. Chromatin-immunoprecipitation-sequencing data from the Encode database revealed that transcription factors like FBJ murine osteosarcoma viral oncogene homolog (Fos), V-Jun avian sarcoma virus 17 oncogene homolog (Jun), signal transducer and activator of transcription 1 and 2 (STAT1, STAT2) interact with sequences located 1,000 base pairs upstream from the transcription start site for the genes encoding *Wnt2, Fzd1, Fzd2*, and *Fzd7* (Table 2), and thus directly controlling the expression of these genes in response to cytokines. Currently, there is no information for the other Wnt or FZD family members.

**Table 2:** **Transcription factors activated by cytokine signaling that interact with 5′ regulatory sequences of genes encoding for Wnt or Fzd receptors**.

Wnt pathway component	Transcription factor associated with inflammation
FZD2	STAT3, AP-1, nuclear factor of activated T cells (NFAT)
WNT3	STAT3, NFκB, NFAT, LEF1
Wnt8	NFAT, NFκB, STAT5
FZD1	IRF, LEF1, NFκB, NFAT, STAT3, AP-1
Wnt1	NFAT, IRF, NFκB
Wnt2	NFAT, LEF
Wnt4	IRF, NFκB
Wnt5	NFAT, IRF, LEF-1, STAT6, STAT5, NFκB
WNT6	STAT6, NFκB
WNT7	NFκB, NFAT, IRF
Wnt10a	STAT6, IRF, NFκB, NFAT, LEF
Wnt10b	STAT5, NFκB, NFAT, IRF, AP-1

*Data obtained from the Encode project. Based on chromatin immunoprecipitation-sequencing data including 1,000 bp upstream from the transcription start site*.

## Wnt3

**Figure 6:**
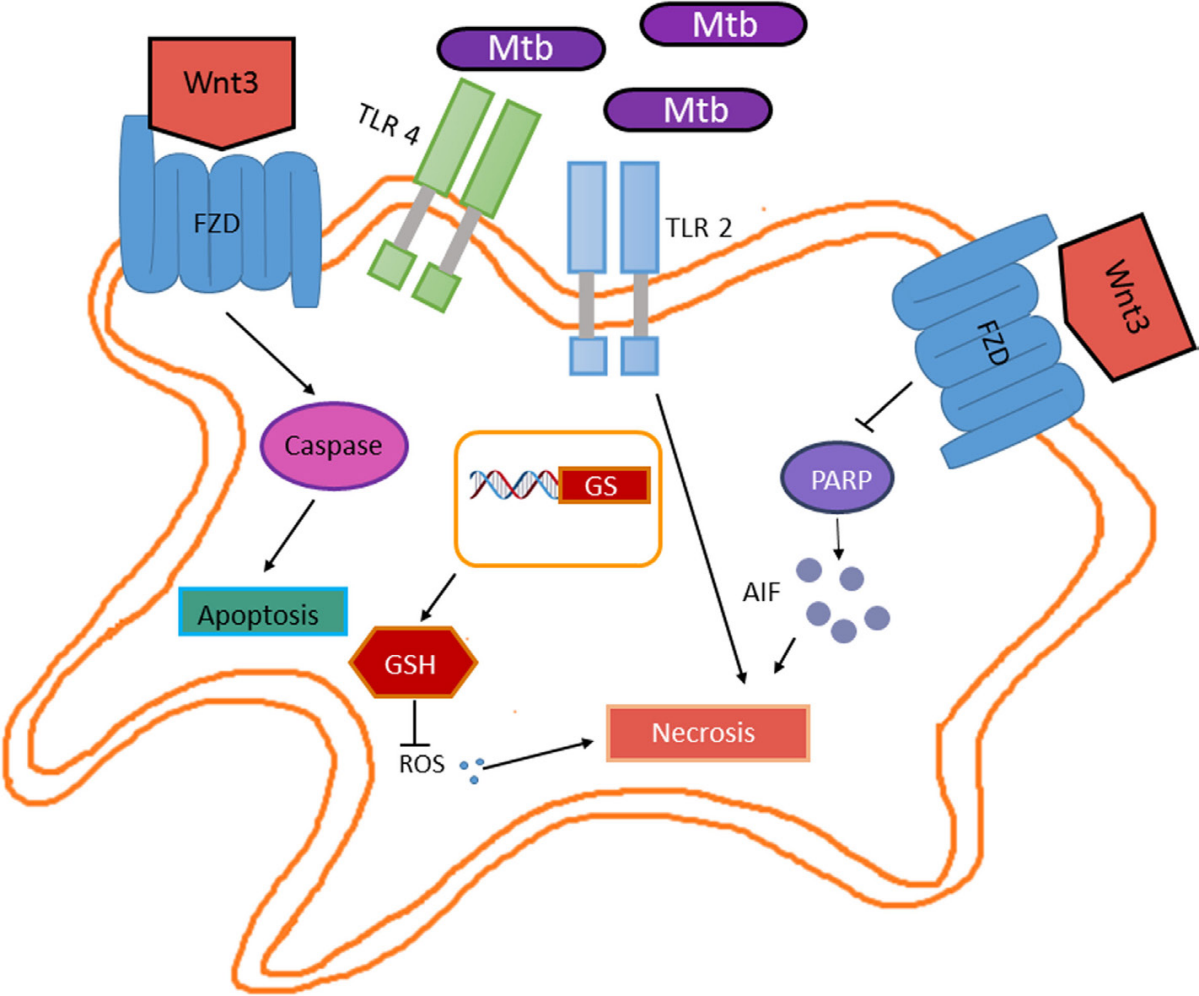
**Wnt3 promotes apoptosis in response to *Mycobacterium tuberculosis* (*M. tuberculosis*) infection**. Wnt3 plays a protective role in the *M. tuberculosis* infection by preventing bacterial dissemination. On one hand, Wnt3 promotes apoptosis in a caspase-dependent manner, while on the other hand inhibits necrosis by promoting glutathione synthase expression (GS) and thus increasing the levels of glutathione (GSH) that in turn results in reduced reactive oxygen species and by inhibiting the PARP/AIF pathway.

Wnt3 is known as a canonical member of the Wnt family. Although monocytes and granulocytes express FZD receptors (FZD1, 5, and 7), when exposed to Wnt3 *in vitro*, only monocytes’ transendothelial migration capacity is reduced (51). This involves the activation of Wnt/β-catenin pathway and increased cell adhesion to endothelial cells (45), hence, suggesting an anti-inflammatory role for Wnt3. However, the Wnt/β-catenin regulated genes involved in enhanced endothelial cell adhesion remain to be identified. In the context of *M. tuberculosis* infection, the role of Wnt3 is controversial. In bone marrow-derived macrophages, *M. tuberculosis* H37rv promotes *Fzd1* receptor gene expression through the TLR-2/MYD88/NFκB pathway; *M. tuberculosis*-induced *Fzd1* expression also involves TNF (52). Interestingly, INFγ enhances Fzd1 levels on the cell surface of *M. tuberculosis-*infected macrophages. According to its proposed anti-inflammatory function, Wnt3 reduced TNF secretion but not *Tnf* gene expression in *M. tuberculosis*-infected macrophages. This effect is mediated through the canonical pathway, since Wnt3 induces β-catenin stabilization and Axin2 expression (52). Additionally, Wnt3 also promotes the expression of Arginase 1 in *M. tuberculosis-*infected macrophages, which has been associated with the anti-inflammatory M2 phenotype (44). In the lung epithelia, Wnt3 is expressed constitutively. Although *M. tuberculosis* infection does not alter Wnt3 expression in the lung epithelia, it promotes *Fzd1* and *Fzd5* gene expression while reducing the expression of *Fzd 3,4, 6–10*, this correlates with reduced β-catenin protein levels and thus β-catenin-dependent gene expression (52). However, whether *in vivo M. tuberculosis*-infected macrophages contain increased levels of the Fzd1 receptor on their cell surface and thus making them susceptible to Wnt3 activation remains to be determined, since the expression levels of the distinct *Fzd* receptors genes were determined using mRNA obtained from total lung homogenates. Nonetheless, the Wnt3 negative effect on TNF release by *M. tuberculosis-*infected macrophages suggests anti-inflammatory functions in murine macrophages (44). Moreover, it has been described that Wnt3 also inhibits IL-6 during mycobacteria infection (44). Interestingly, Wnt3 enhances apoptotic cell death in RAW 264.7 macrophage infected with *M. bovis* bacillus Calmette–Guerin (BCG), through a mechanism involving the regulation of pro-apoptotic and antiapoptotic protein levels. *M. bovis* BCG-infected macrophages showed increased levels of the pro-apoptotic protein Bax and reduced levels of the antiapoptotic protein Mcl-1 in response to Wnt3 exposure (46). Thus, by inducing apoptosis of infected macrophages, Wnt3 prevents bacterial dissemination. Consistent with this idea, another study showed that Wnt3 reduced the levels of reactive oxygen species (ROS) resulting from BCG infection, which correlates with the increase in glutathione levels (Figure 6) (46). However, whether the elevated glutathione levels induced by Wnt3 result from an increase in the enzymatic activity of the enzymes involved in glutathione synthesis (gamma-glutamylcysteine synthetase and glutathione synthetase) or from increased expression of the genes encoding these enzymes, remains to be determined. Together, these results suggest that at early stages of the infection, Wnt3 prevents the dissemination of infectious mycobacteria by promoting apoptosis and reducing necrosis of infected macrophages. However, Wnt3 promotes the differentiation toward an M2 anti-inflammatory phenotype of the infected macrophages that survive, thus attenuating the immune response against *M. tuberculosis* and therefore promoting TB.

## Wnt5

Genomic analyses have shown that macrophages and dendritic cells upregulate Wnt5a mRNA levels when exposed to pathogens (52, 53). Also, during the differentiation process of monocytes to macrophages or dendritic cells, granulocyte macrophage colony-stimulating factor and IL-4 promote *Wnt5a* gene expression (51). In Raw 264.7 macrophages, *M. tuberculosis* infection increases Wnt5 protein levels. Accordingly, *WNT5A* and WNT5A receptor *FZD5* mRNA levels are induced in human macrophages in response to *M. tuberculosis* and *M. avium* infection (19). *M. tuberculosis*-dependent *WNT5a* expression requires TLR2 signaling and NFκB activation; however, whether *M. tuberculosis* uses the same signaling pathway to induce *FZD5* gene expression remains to be confirmed. Interestingly, human peripheral blood mononuclear cells expressing *WNT5A* and *FZD5* mRNA were detected in granulomatous lesions in the lungs of *M. tuberculosis*-infected patients (48). Given that the IL-12 production by mycobacterium-infected macrophages results from a Wnt5a autocrine loop and that activated T cell express FZD5 at the mRNA and protein levels (48), these experimental evidence indicates that INFγ production by mycobacterial antigen-stimulated T cells is induced not only by IL-12 but also by the interaction of WNT5A with FZD5 receptor expressed on the T cell surface. Whether the WNT5A-mediated INFγ production in T cells involves the non-canonical Wnt pathway remains to be determined. Nonetheless, the paramount role of INFγ in the adaptive immune response against *M. tuberculosis* suggest that Wnt5a produced by mycobacterium-infected macrophages also play a key role against *M. tuberculosis* control. However, recently, it was shown that *M. bovis* BCG prevents INFγ-induced macrophage autophagy through a mechanism that involves Wnt5a, which enhances the expression of ALOX5 and ALOX15 and thus the production of anti-inflammatory lipids (54). Together, these results place Wnt5a in the center of the fine balance that is required for maintaining the granuloma to avoid bacterial dissemination and also to prevent bacterial destruction. Whether Wnt5a is also required for granuloma formation remains to be determined.

Although the mechanism by which mycobacteria promotes *Wnt5* expression remains largely unknown, recently, it has been demonstrated that dectin-1 activation through different dectin-1 ligands, including mycobacteria, results in Wnt5 expression (54). Upon dectin-1 engagement, activation of the tyrosine kinase SYK and ROS production lead to the inactivation of GSK3 resulting in β-catenin stabilization and thus Wnt5 expression (19). In addition to the functions described above, Wnt5 through an autocrine loop activates the non-canonical Wnt pathway, resulting in the activation of calcium-activated calmodulin kinase (Ca^++^/CAMKII) pathway and the expression of the protein inhibitors of activated STAT (PIAS1) and suppressor of cytokine signaling (SOCS1) expression (54). Overexpression of PIAS1 and SOCS1 in macrophages dampens TLR-induced pro-inflammatory response by reducing the protein levels of pivotal TLR adaptors such as IRAK1, IRAK4, and Myd88 (19). Accordingly, *in vivo* activation of dectin-1 receptor with pathogenic or fungi ligands resulted in an increased mycobacteria burden and a concomitant decrease in TLR-triggered pro-inflammatory cytokines (54). This might also involve impaired phagosome–lysosome fusion and bacterial destruction, since activation of the non-canonical Wnt pathway by Wnt5 involves Ca^++^ (37, 38), which is required for coronin-1 recruitment to the phagosome and calcineurin activation to prevent phagosome–lysosome fusion (55).

## Wnt6

In a recent study, Schaale and coworkers showed that Wnt6 is expressed in granulomatous lesions in the lung of *M. tuberculosis*-infected mice (50). Wnt6 expression in response to mycobacteria is TLR-Myd88-NFκB-dependent. Interestingly, foamy macrophage-like cells are the principal source of Wnt6. Secreted Wnt6 acts as a paracrine signal on neighboring macrophages to promote proliferation and macrophages polarization toward an anti-inflammatory M2 like phenotype (50). The Wnt6-induced signal transduction driving macrophages proliferation and polarization does not involve β-catenin, but relay on G protein-dependent ERK activation and c-Myc expression, a pivotal regulator of cell proliferation. These observations are consistent with another study showing a key role for c-Myc in IL-4-induced alternative activated phenotype in human macrophages (56). In this scenario, c-Myc directly regulates the expression of genes associated with the M2 phenotype like scavenger receptor class B member 1, ALOX15, and mannose receptor, C-type 1 and indirectly regulates the expression of CD206 while strengthening IL-4 signaling by upregulating STAT6 and PPARγ expression (56). It remains to be determined whether these molecules are also involved in M2 polarization resulting from Wnt6 exposure. Nonetheless, these results point out an important role for Wnt6 in promoting macrophages alternative activation within the granulomatous lesions, thus providing *M. tuberculosis* a granuloma with an anti-inflammatory microenvironment that ensures its survival.

## Another Wnt Homolog

Besides the roles of Wnt3, Wnt5, and Wnt6 in *M. tuberculosis* infection discussed above, the role of other Wnt homologs is unknown. In a recent study where Wnt6 was identified as a molecule expressed exclusively in granulomatous lesions, Schaale and coworkers monitored the expression of all Wnt homologs in mice infected with *M. tuberculosis* (50). They found that mRNA levels of most Wnt homologs were significantly reduced in the course of infection (Wnt2, Wnt2b, Wnt3a, Wnt4, Wnt5, Wnt7a, Wnt8a, and Wnt10b), while other homologs were upregulated (Wnt1, Wnt6, and Wnt10a) (50). The reduction in the expression levels of the majority of the Wnt homologs during mycobacterial infection is consistent with a study that indicates that FZD family of receptors and the Wnt/β-catenin target gene Axin2 are significantly downregulated in the lungs of *M. tuberculosis*-infected mice (44).

In a genetic study made in a Chinese population, 25 polymorphisms (SNPs) in the Wnt pathway related to TB risk were evaluated. Five polymorphisms were associated with TB susceptibility: secreted frizzled-related protein (SFRP1) rs4736958, CTNNB1 (catenin-β1), rs9859392, rs987055, and rs3864004 showed decreased TB risk, meanwhile, SFRP1 rs7832767 was associated with increased risk (57, 58). According to these results, the authors provide evidence that Wnt pathway polymorphisms may influence TB susceptibility and host immune response to *M. tuberculosis*, suggesting that these variations could serve as novel markers to identify the risk and susceptibility to develop TB (57, 58).

## Nucleotide-Binding Oligomerization Domain (NOD2) Mediates the Activation of the Wnt Pathway in Response to *M. tuberculosis* Infection?

**Figure 7:**
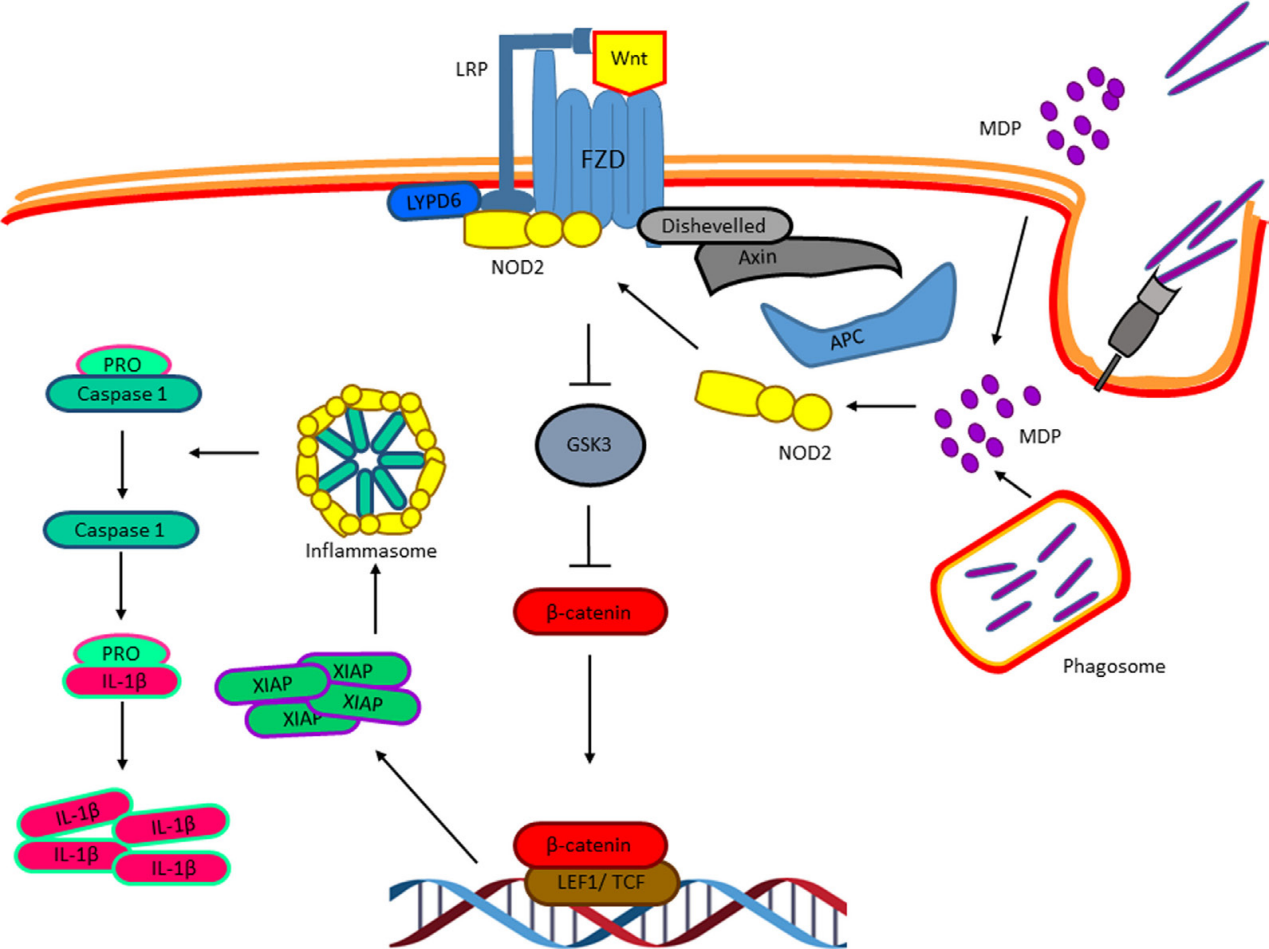
**Nucleotide-binding oligomerization domain 2 (NOD2) and the activation of the Wnt pathway in response to *Mycobacterium tuberculosis* infection**. Muramyl dipeptide, derived from the Mycobacterial cell wall at early phases of the macrophages’ infection, triggers NOD2 translocation to the membrane where it interacts with the Ly6/PLAUR domain-containing protein 6, which has been previously shown to be recruited to the Wnt receptor complex and promote Wnt signaling resulting in β-catenin nuclear translocation and the expression of the X chromosome-linked inhibitor of apoptosis, that in turn activates the NLRP3 inflammasome resulting in IL-1β maturation.

So far, we have discussed distinct experimental evidences that clearly indicate an important role of the Wnt factors on the biology of *M. tuberculosis*-infected macrophages and that *M. tuberculosis* modulates the expression of these factors and their receptors to ensure successful infection. However, whether *M. tuberculosis* triggers the Wnt pathway directly, is still an open question. The intracellular pathogen sensor NOD2, which is activated by bacterial cell wall components like peptidoglycan, specifically by muramyl dipeptide (MDP), has been shown to be activated during macrophage infection with *M. tuberculosis* and to synergize with the TLR signaling pathway to promote the production of pro-inflammatory cytokines (59–61). Interestingly, last year, it was suggested that the activation of NOD2 by MDP results in the activation of the Wnt pathway (62). In the presence of MDP, NOD2 is translocated to the membrane where it interacts with the Ly6/PLAUR domain-containing protein 6, which has been previously shown to be recruited to the Wnt receptor complex and promotes Wnt signaling (63) that results in β-catenin nuclear translocation and the expression of the X chromosome-linked inhibitor of apoptosis, that in turn activates the NLRP3 inflammasome leading to IL-1β maturation and secretion (62). Thus, it is possible that MDP, derived from the mycobacterial cell wall at early phases of the macrophages infection, triggers NOD2 that activates the Wnt pathway directly even in the absence of Wnt factors (Figure 7) to promote caspase-1 activation and IL-1β production, and probably some of the biological effects attributed to specific Wnt factors discussed above. Nonetheless, this hypothesis remains to be tested.

## *M. tuberculosis* Fine Tuning of the Wnt Pathway through microRNAs (miRNAs) Regulation

**Figure 8:**
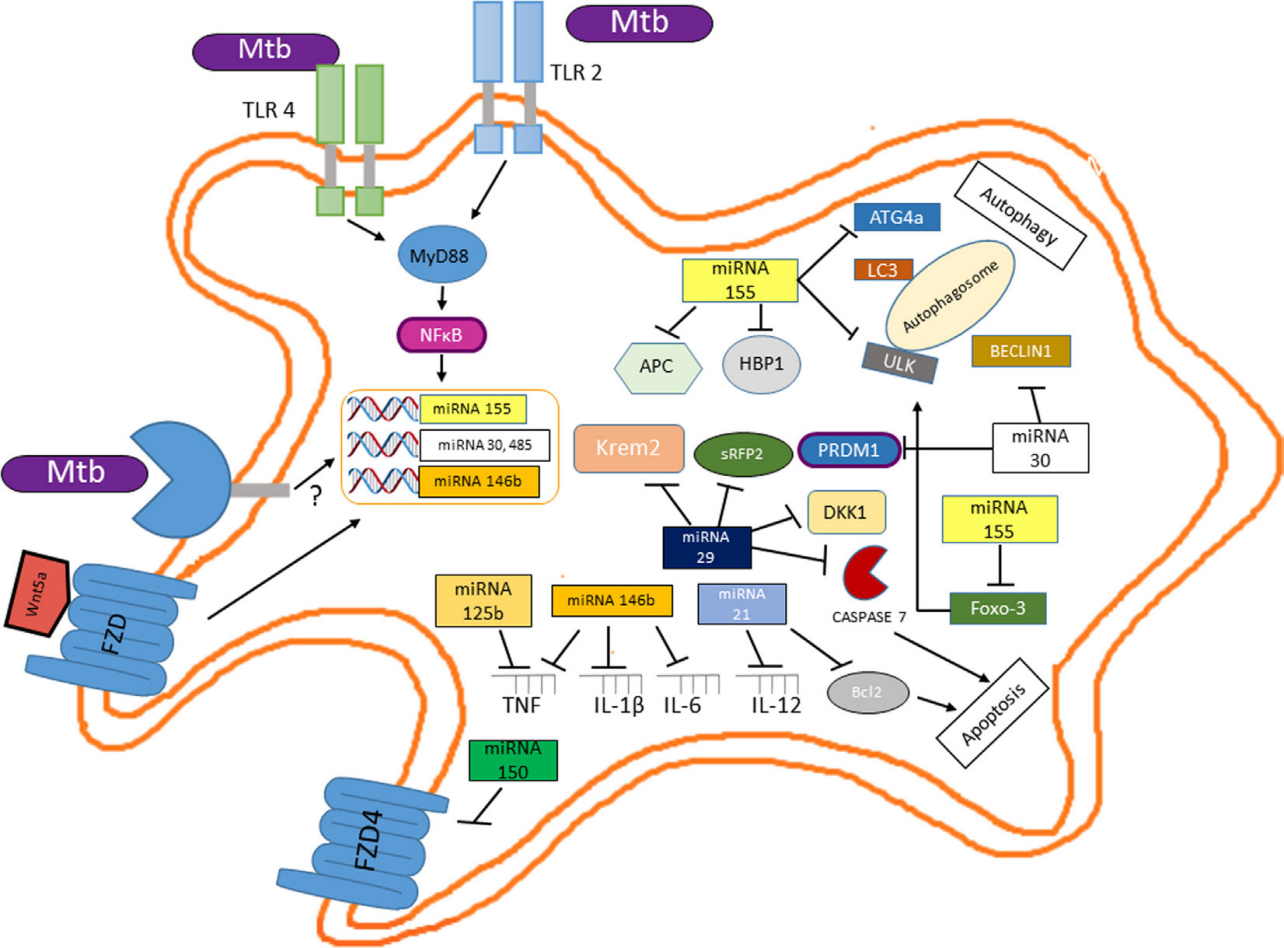
**Mutual regulation between miRNAs and the Wnt pathway determine the survival of *Mycobacterium tuberculosis* (*M. tuberculosis*)-infected macrophage and the profile of the inflammatory response**. Upon interaction of *M. tuberculosis* with TLR2 and TLR4, NFκB activation leads to increased miR155 levels which, besides its negative effect on autophagy and apoptosis, through the negative modulation of key inhibitors of the Wnt pathway (APC and HMG-box transcription factor 1) leads to β-catenin/TCF-mediated expression of miR2, miR125b, miR146b (a target of the Wnt5a signaling pathway), and miR29, thus wiping off the pro-inflammatory response. Additionally, miR29 further enhances Wnt signaling by targeting distinct set of Wnt negative regulators (Dikkopf-1, Kremen2, and sFRP2).

A novel layer of complexity in *M. tuberculosis* regulatory networks controlling host infection, survival, and successful infection has recently emerged with the discovery that *Mycobacterium* regulates the expression of different miRNAs (64). miRNAs regulate the protein abundance in any given cell at any given time, thus influencing many cellular processes including differentiation, proliferation, apoptosis, DNA methylation, DNA repair, and provide anti-inflammatory or pro-inflammatory stimuli (65, 66). According to this, it is not surprising to find that *M. tuberculosis* positively modulates the expression of miRNAs that target key molecules involved in the destruction of the pathogen like apoptosis or autophagy and the setting of protective cytokine profiles while decreasing the expression of miRNAs whose targets are molecules involved in the negative control of these biological processes [reviewed in Ref. (67, 68)]. Although we know that the miRNA profile expressed by infected macrophages depends on the infecting *Mycobacterium* species (64, 69, 70), the virulence of the strain (71), or the stage of the disease (69), the role of those miRNAs and the signaling pathway that control their expression remains to be elucidated. Nonetheless, at early stage of infection, inhibition of autophagy and apoptosis and the control of the inflammatory response by *M. tuberculosis* involve different miRNAs, some of which could mediate their effect through the induction of the Wnt signaling pathway or be regulated directly by this pathway (Figure 8). At the onset of infection, miR155 expression increase in *M. tuberculosis-*infected macrophages (72). The induction of this miRNA may be mediated by the activation of NFκB (73); thus, miR155 could impair autophagy in *M. tuberculosis-*infected macrophages through its interaction with the 3′ UTRs of different mRNAs encoding for key proteins like ULK1, involved in the initiation phase; ATG14, part to the BECLIN1 complex that promotes phagophore nucleation; ATG5, ATG3, LC3, and GABARAPL1, key players in the elongation and maturation steps; and FOXO3, a transcription factor regulating the expression of autophagy-related genes, including MAP1LC3, ULK1, ATG14, and GABARAPL1 (74). Additionally, miR30a, another miRNA expressed in infected macrophages, through its interaction with BECLIN1 (75) could also impair autophagy. Interestingly miR155, through its negative effect on APC (76) and HMG-box transcription factor 1 (77), a strong Wnt pathway suppressor and miR30a by blocking PRDMI (78) could induce β-catenin/TCF-mediated gene expression, and thus regulate the expression of additional miRNAs, like miR21 and miR125b (79) and miR146b, which is positively regulated by Wnt5a (80) and miR29 (81, 82). miR21 by reducing BCL2 protein levels (83) and miR29 by targeting caspase-7 (84) might prevent apoptosis of infected macrophages. Additionally, miR29 could also further enhance the Wnt signaling pathway by targeting the negative regulators of Wnt signaling, Dikkopf-1, Kremen2, and secreted frizzled-related protein 2 (sFRP2) (81, 82). While miR146b, by targeting IL-1β, IL-6, IRAK1, TRAF6, TNF, TLR4, NFκB1 (85), and miR21 by negatively regulating IL-12 (86) damp the inflammatory response. Additionally, given that the lung epithelium constitutively produces Wnt3 and that the canonical Wnt pathway promotes miR125b expression (74), at early stages of *M. tuberculosis* infection, miR125b could also contribute to attenuate the inflammatory response by targeting the TNF 3′ UTR (87). Together, these results strongly indicate that, at early stages of infection, the Wnt pathway controls key macrophage functions by modulating miRNAs expression that allows *M. tuberculosis* survival.

**Figure 9:**
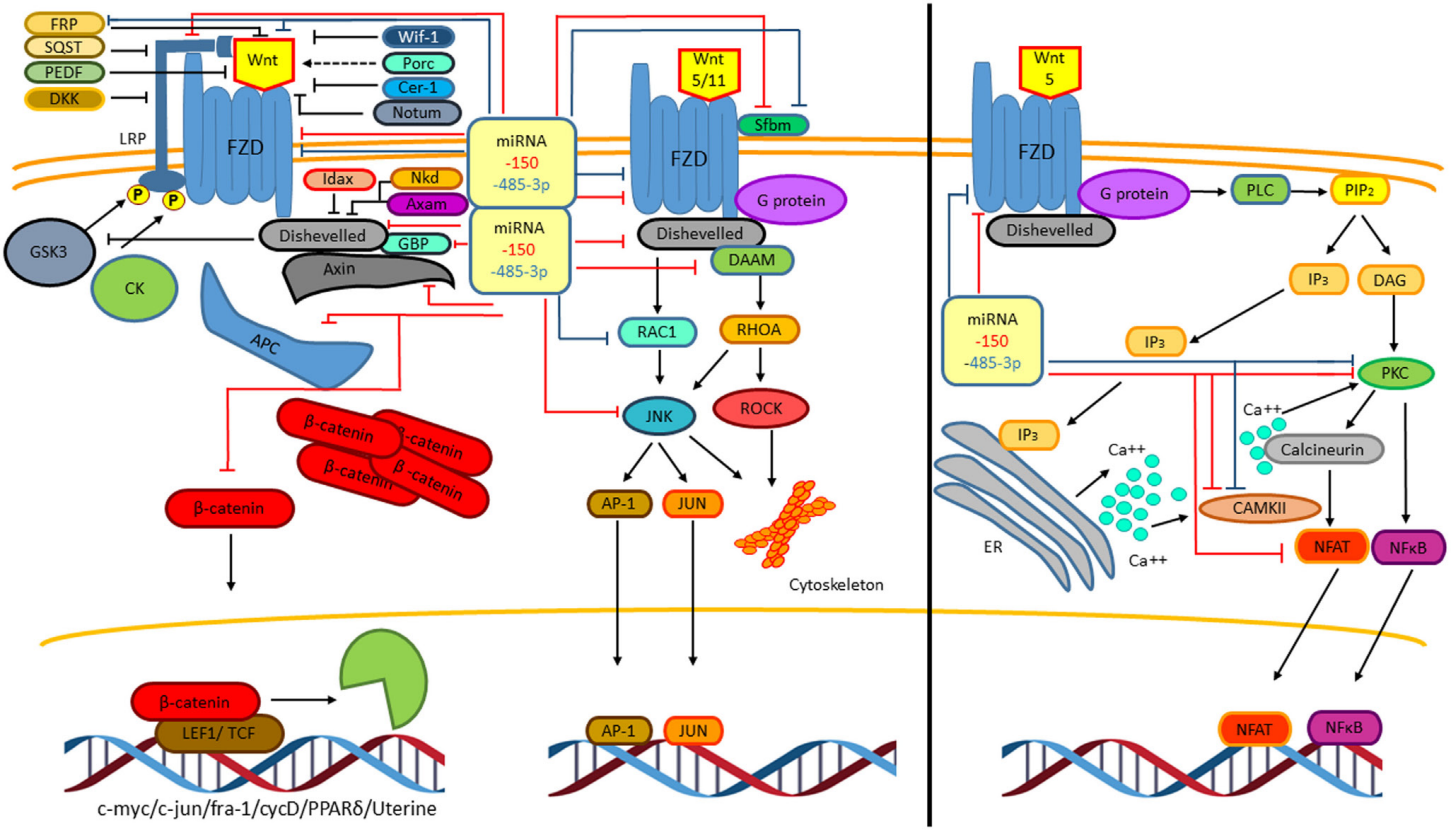
**The Beijing *Mycobacterium tuberculosis* (*M. tuberculosis*) strains regulate the Wnt pathway during macrophage infection of healthy individuals or that of macrophages from patients with active tuberculosis by enhancing miR485 and reducing miR150 levels, respectively**. An enrichment pathway analysis performed in the WebGestalt platform using the Kyoto Encyclopedia of Genes and Genomes indicates that the miRNA485 potentiates the canonical Wnt pathway through negatively regulating protein levels involved in the inhibition of these pathways, while by controlling the protein levels of key signaling molecules might inhibit the non-canonical Wnt pathway. In contrast, miR150 has clear inhibitory effect on both the canonical and non-canonical Wnt pathway. Thus, through differentially regulating the expression of these two miRNAs, the Beijing *M. tuberculosis* strains promote the Wnt canonical pathway.

A recent study points out that different *M. tuberculosis* clinical strains induced specific macrophage miRNAs expression patterns (69). Macrophages infected with Beijing *M. tuberculosis* strains showed reduced expression of 13 miRNAs compared with macrophages infected with non-Beijing *M. tuberculosis* strains. Interestingly, only miR485 showed increased levels in the Beijing infected macrophage when compared with non-Beijing infected macrophages. Our bioinformatics analysis indicates that this miRNA has potential binding sites with thermodynamic values that allow specific miRNA/mRNA interactions within the 3′ UTR of mRNAs encoding for proteins involved in the Wnt pathway (Table 3). A gene ontology analysis revealed that through negatively modulating the levels of the SFRP1 and those of the Strabismus 1 (STB1), miR485 might positively regulate the canonical Wnt pathway. In contrast, miR485 might inhibit the non-canonical Wnt pathways by negatively modulating FZD4, RAC-1, and PKC (88) (Figure 9). The previous data indicate that the Beijing *M. tuberculosis* clinical strains upregulate the canonical Wnt pathway by inducing miR485 expression, to modulate the macrophage response in order to ensure a successful infection.

**Table 3:** **Predicted^a^ miRNA485 target genes**.

Target gene	Full name	Potential binding sites^b^	ΔΔ*G*^c^	Validated
SMAD3	SMAD family member 3	1	–10.15	No
FZD4	Frizzled family receptor 4	1	–9.49	No
RAC1	Ras-related C3 botulinium toxin substrate 1	1	−8.85	No
PPP2R5C	Protein phosphatase 2, regulatory subunit B′, γ	1	−15.08	No
SFRP1	Secreted frizzled-related protein 1	1	−8.96	No
SMAD4	SMAD family member 4	1	−9.71	No
CAMK2G	Calcium/calmodulin-dependent protein kinase II γ	1	−8.41	No

*^a^Target prediction was performed using PITA (https://genie.weizmann.ac.il) and TargetScan (http://www.targetscan.org) platforms*.

*^b^Only the potential interaction sites with a ΔΔG ≤ 8 were considered*.

*^c^ΔΔG values were calculated using PITA*.

On the other hand, from the miRNAs negatively regulated by the Beijing *M. tuberculosis* clinical strains in both THP-1 derived macrophages or in peripheral blood-derived macrophages obtained from TB patients with active disease, miR150 showed the most prominent reduction (69). Once again, our bioinformatic analysis indicated that, this miRNA has numerous potential targets in the Wnt pathway (Table 4). According to thermodynamic values indicating strong possibilities for specific miRNA/mRNAs interactions, from the predicted targets, FZD4 has been shown to be a *bonafide* miR150 target (89). The pathway analysis of the potential miR150 targets indicates that this mRNA might negatively regulate both the canonical and non-canonical Wnt pathways (Figure 9). Together, these results suggest that the upregulation of miR485 and the negative regulation of miR150 levels are key events triggered by Beijing *M. tuberculosis* clinical strains to sustain the activation of the canonical Wnt pathway during macrophage infection of healthy individual and that of individual with active disease. However, whether the opposite regulation of these two miRNAs by the Beijing *M. tuberculosis* is hallmark of the virulence phenotype remain to be determined. Nonetheless, it is clear that the regulation of miRNAs expression by *M. tuberculosis* constitutes a novel mechanism to modulate the Wnt pathway during infection and also that through modulating the levels of specific miRNAs, the Wnt pathway ensures the macrophage infection with *M. tuberculosis* and its survival by promoting a anti-inflammatory environment.

**Table 4:** **Predicted^a^ miRNA150 target genes**.

Target gene	Full name	Potential binding sites^b^	ΔΔ*G*^c^	Validated
MAPK8	Mitogen-activated protein kinase 8	1	−12.38	No
FZD4	Frizzled family receptor 4	1	−8.94	Yes
DAAM2	Disheveled-associated activator of morphogenesis 2	1	−9.87	No
PRKX	Protein kinase, X-linked	2	−12.68, −9.81	No
FRAT2	Frequently rearranged in advanced T-cell lymphomas 2	2	−19.27, −8.04	No
DVL3	Disheveled, dsh homolog 3	1	−8.09	No
LRP5	Low-density lipoprotein receptor-related protein 5	1	−16.42	No
APC	Adenomatous polyposis coli	1	−11.51	No
PRKACA	Protein kinase, cAMP-dependent, catalytic α	1	−12.16	No
NFAT5	Nuclear factor of activated T-cell 5	2	−12.43, −8.84	No
FZD7	Frizzled family receptor 7	1	−13.9	No
CREBBP	CREB binding protein	1	−10.29	No
CCND1	Cyclin D1	1	−13.9	No
PRICKLE2	Prickle homolog 2	3	−16.17, −15.12, −11.45	No
NFATC4	Nuclear factor activated of T-cells, cytoplasmic, calcineurin-dependent 4	1	−8.07	No
ROCK1	Rho-associated, coiled-coil containing protein kinase 1	2	−11.35, −8.53	No
CCND2	Cyclin D2	1	−9.2	No
CAMK2G	Calcium/calmodulin-dependent protein kinase II γ	1	−10.98, −9.19	No
DVL2	Disheveled, dsh homolog 2	1	−11.46	No

*^a^Target prediction was performed using the PITA (https://genie.weizmann.ac.il) and TargetScan (http://www.targetscan.org) platforms*.

*^b^Only the potential interaction sites with a ΔΔG ≤ 8 are were considered*.

*^c^ΔΔG values were calculated using PITA*.

## Concluding Remarks

In the course of *M. tuberculosis* infection, several pathways are induced to promote inflammation to eliminate the pathogen and to attenuate the inflammatory response to avoid tissue damage. The data discussed above strongly suggest that the Wnt signaling pathway plays a key role at different stages of TB development, including *M. tuberculosis* survival in the macrophages, modulation of the inflammatory response, and later on granuloma formation and to control the adaptive immune response. However, further research is required to determine: (i) whether *M. tuberculosis* can directly activate the Wnt signaling pathway upon macrophage infection; (ii) which of the biological effects attributed to cytokines are mediated by Wnt factors; (iii) whether virulent strains are more dependent on the Wnt pathway to ensure infection; and (iv) define how the different miRNAs expressed in response to *M. tuberculosis* infection modulate the Wnt signaling pathway, among other important processes. Efforts to determine the mRNA and miRNAs profile of infected macrophages obtained from mouse or human granulomas using deep sequencing will provide a clear picture of which Wnt factors, FZD receptors, and Wnt-dependent genes are expressed among them miRNAs, the pathway targeted by these miRNAs and if all of this depend on the *M. tuberculosis* strain, the virulence or drugs resistance. This information may identify candidates for the development of new therapeutic strategies to promote mycobacterial destruction in the granuloma. Likewise, future clinical studies may provide new insights for therapeutic applications of Wnt factors.

## Author Contributions

TV participated in the conception of the work, gathered information, and wrote the first draft of the paper. EM-P gathered information, performed bioinformatic analysis, and participated in the edition of the final draft. RM-B gathered information, performed artwork, and participated in the edition of the final draft. AU-A gathered information and performed the gene ontology and pathway enrichment analysis for the miRNA section. LP-M participated in the conception of the work, gathered information, and participated in the edition of the final draft. GP-A participated in the conception of the work, gathered information, and participated in the edition of the final draft.

## Conflict of Interest Statement

The authors declare that the research was conducted in the absence of any commercial or financial relationships that could be construed as a potential conflict of interest.
